# Parental effects influence life history traits and covary with an environmental cline in common frog populations

**DOI:** 10.1007/s00442-020-04642-8

**Published:** 2020-04-10

**Authors:** Piotr K. Rowiński, Anssi Laurila, Karl Gotthard, Will Sowersby, Martin I. Lind, Alex Richter-Boix, Simon Eckerström-Liedholm, Björn Rogell

**Affiliations:** 1 Department of Zoology, Stockholm University, 106 91 Stockholm, Sweden; 2 Animal Ecology, Department of Ecology and Genetics, Uppsala University, 752 36 Uppsala, Sweden; 3 Present Address: Department of Aquatic Resources, Institute of Freshwater Research, Swedish University of Agricultural Sciences, 178 93 Drottningholm, Sweden

**Keywords:** Maternal effects, Phenology, Local adaptation, Temperature, Trade-offs

## Abstract

Across latitudinal clines, the juvenile developmental rates of ectotherms often covary with the length of the growing season, due to life-history trade-offs imposed by the time-constrained environments. However, as the start of the growing season often varies substantially across years, adaptive parental effects on juvenile developmental rates may mediate the costs of a delayed season. By employing a meta-analysis, we tested whether larval developmental rates across a latitudinal cline of the common frog (*Rana temporaria*) are affected by fluctuating onsets of breeding, across years. We predicted that larval developmental rate will be inversely related to the onset of breeding, and that northern populations will be more prone to shorten their developmental rate in response to late breeding, as the costs of delayed metamorphosis should be highest in areas with a shorter growing season. We found that the larval period of both northern and southern populations responded to parental environmental conditions to a similar degree in absolute terms, but in different directions. In northern populations*,* a late season start correlated with decreased development time, suggesting that the evolution of parental effects aids population persistence in time-constrained environments. In southern populations, late season start correlated with increased development time, which could potentially be explained as a predator avoidance strategy. Our findings suggest that local ecological variables can induce adaptive parental effects, but responses are complex, and likely trade-off with other ecological factors.

**Electronic supplementary material:**

The online version of this article (10.1007/s00442-020-04642-8) contains supplementary material, which is available to authorized users.

## Introduction

Across taxa, environmental parental effects occur when environmental factors experienced by parents affect the phenotype of their offspring, independent of the parent genetic contribution (Bernardo 1996; Mousseau and Fox 1998a). Environmentally induced parental effects may be adaptive, if they allow parents to modify offspring phenotypes to increase offspring fitness, in response to reliable environmental cues, such as photoperiod or temperature (Rossiter 1996; Marshall and Uller 2007; Uller et al. 2013). Consequently, parental effects are considered to be an important part of evolutionary processes (Kirkpatrick and Lande 1989; Mousseau and Fox 1998b; Räsänen and Kruuk 2007; Mousseau et al. 2009). However, relatively few studies have clearly demonstrated the adaptive value of parental effects (Uller et al. 2013). Potentially due to the subtle nature of parental effects, especially if within generational environmental influences dominate offspring phenotypes, in comparison to between generational influences (Uller et al. 2013; but see also Donelan and Trussell 2018). Moreover, adaptive maternal effects are expected to evolve when environmental conditions differ across generations, when there are reliable cues about offspring environment, and if the cost of cue detection by the offspring is low, and if there is no or low levels of intergenerational conflict to maximize fitness (Mousseau and Fox 1998b; Marshall and Uller 2007; Kuijper and Hoyle 2015; Dury and Wade 2019). However, despite clear theoretical predictions for parental effects to evolve in response to local environmental conditions, there is limited empirical knowledge on the evolution of parental effects across varying environmental conditions (but see Dey et al. 2016; Lind et al. 2019).

One situation where maternal effects have been suggested as a key adaptive trait, is across environmental clines (Mousseau and Dingle 1991; Johnston and Leggett 2002). Across environmental clines, developmental time-constraints often occur at higher latitudes, and altitudes, or with low precipitation levels, which tend to select for localized adaptation (Berven 1982; Laugen et al. 2003; Rogell et al. 2009; Eckerström-Liedholm et al. 2017). For example, in ectotherms, low temperatures decrease the activity of biochemical and physiological processes (Zuo et al. 2012), resulting in slow developing phenotypes that risk failing to complete their life cycle, or to attain a size necessary to survive winter. As these plastic responses incur costs, natural selection often selects on higher intrinsic developmental rates to counteract the disadvantageous plastic responses induced by low temperatures (e.g., “counter-gradient variation”, Conover and Schultz 1995). Counter-gradient variation has been reported in a wide variety of ectotherms, such as fish, amphibians, reptiles, mollusks, and insects (e.g., Conover and Present 1990; Laugen et al. 2003; Du et al. 2010; Li et al. 2018; Trussell 2002; Arnett and Gotelli 1999; but see also Tang et al. 2017). Why all populations do not always grow fast, has been attributed to life-history trade-offs caused by ecological (Laurila et al. 2006, 2008) or physiological factors (Conover and Present 1990; Levinton 1983). For example, along a latitudinal gradient of common frog (*Rana temporaria*) populations, intrinsic developmental rate increases with increasing latitude (Laugen et al. 2003). However, the faster development of northern *R. temporaria* entails costs, such as the requirement for higher levels of risk-taking behaviors necessary for obtaining the resources to fuel fast development, which in turn increases mortality risk, by increasing exposure to predators (Laurila et al. 2006, 2008). In addition, individuals with high growth rates are expected to have higher energetic requirements for bodily maintenance (Conover and Present 1990), and may therefore be more severely affected by sub-optimal food availability, compared to individuals with slower growth (Levinton 1983). In *R. temporaria*, tadpoles from northern populations indeed need higher-quality food to realize their developmental potential, and they also have more efficient digestion than tadpoles from southern populations (Liess et al. 2015).

The existence of counter-gradient variation implies that time-limitations select on the developmental rates of ectotherms (Palo et al. 2003; Richter-Boix et al. 2010). Importantly, time-limitations may occur due to spatial or temporal variation in temperature, which influences length of the growing season. In such cases, adaptive parental effects, in addition to phenotypic plasticity, may allow parents to match the phenotypes of their offspring to the conditions experienced under each specific year. Indeed, earlier studies have shown that an experimental delay in parental reproduction, induces presumably adaptive parental effects in the moor frog (*Rana arvalis*), by decreasing larval periods, and by increasing growth rate and mass at metamorphosis (Richter-Boix et al. 2014; Orizaola et al. 2016). Further, northern populations of *R. temporaria* shortened larval development during a late breeding season, in comparison to an earlier breeding season (Orizaola et al. 2013), while more southern populations increased their mass at metamorphosis and growth rate when their reproduction was postponed artificially (Lindgren and Laurila 2010). However, to assess the importance of these parental cues, under natural conditions, a broader investigation is required, spanning multiple years and populations. In addition, as parental effects presumably evolve as a means to cope with variation in time-constrained conditions, it seems likely that parental effects should be contingent on climate-related spatial clines (Mousseau and Dingle 1991; Orizaola et al. 2013; Richter-Boix et al. 2014). In areas with only a short window of opportunity for larval development, any costs incurred by either fast development or growth (Johansson et al. 2001; Janssens and Stoks 2018) are likely to be outweighed by the benefits of reaching a specific body size before the end of the growing season. Therefore, time-constrained northern populations may be more prone to display adaptive parental effects by shortening development during breeding seasons that begin late. Alternatively, northern populations may already be at the limits of their physiological capacity for fast development, leaving little scope for any additional parental effects to influence developmental rates (Lankford et al. 2001; Gillooly et al. 2002).

Here, using published data, we tested whether the expression of adaptive parental effects in respect to delayed reproduction is latitude-dependent in *R. temporaria* populations along Sweden (between latitudes of 55° N and 69° N—1500 km across). We investigate how the timing of reproduction influences the expression of paternal effects on populations along an environmental cline, by analyzing 11 data-sets, where either breeding adult frogs were captured for laboratory crosses or eggs were collected immediately after been laid in the wild. The studies used in this meta-analysis, controlled for any external environmental cues by rearing larvae under a common-garden design, which ensured that any observed changes in larval life history traits were due to parental, and not offspring, environmental conditions. We tested if the length of the larval period was affected by breeding delay (difference between onset of spring and mean onset of spring) during the period prior to breeding. We predicted that (1) a later induction of breeding (onset of spring) should decrease length of growing season, and hence correlate with increased rates of larval development, and that (2) this response should be stronger in northern populations, as the costs of delayed metamorphosis should be highest in areas with short growing season.

## Materials and methods

### Study species

*Rana temporaria* is a common and wide-spread anuran in Europe, occurring in a variety of habitats, including tundra, forests, swamps, peatlands, fields, steppe and gardens (Kuzmin et al. 2009). The onset of breeding occurs early in the spring after the ice melts, and depends on both temporal (temperature) and spatial (latitude and altitude) climate-related conditions (Phillimore et al. 2010). *Rana temporaria* populations have been extensively studied across latitudinal gradient in Sweden and northern Finland during the last 20 years (Table 1). See Online Resource 1 for more detailed species information.

**Table 1:** Overview of the data used in the analysis

Data used in the following studies	Collection year	Populations (breeding ponds) sampled (names as in the studies)	Latitudes	Temperature in laboratory (°C)	Density (number of tadpoles per volume of water in liters)	Photoperiod in laboratory (hours light/dark)	Sample size (populations added)	Food in laboratory	Location of experimental procedures	Artificial crosses/eggs collected
Merilä et al. (2000)	1998	Lund, Kiruna	South, North	22	1/0.7	16/8	18	3:1 mixture of rabbit pellets and aquarium fish flakes	Uppsala	South—artificial crosses, North—eggs collected
Palo et al. (2003), Laugen et al. (2003)	1998	Lund, Kilpisjärvi	South, North	18	1/0.9	16/8	172	3:1 mixture of rabbit pellets and aquarium fish flakes	Uppsala	Artificial crosses
Laurila et al. (2002)	1999	Tvedora, Esrange	South, North	21	1/0.75	18/6	270	3:1 mixture of rabbit pellets and aquarium fish flakes	Uppsala	Artificial crosses
Laurila et al. (2001)	2000	Tvedora, Kilpisjärvi	South, North	20	1/0.9	16/8	40	Lightly boiled spinach	Uppsala	Artificial crosses
Laurila et al. (2008)	2001	Kabusa, Barsjon, Stora Almby, Söderfors, Mjosjö, Jukkasjärvi, Karesuando	South, Mid South, Mid North, North	18	10/12	16/8	70	Lightly boiled spinach	Uppsala	Eggs collected
Merilä et al. (2004)	2001	S1, S2, S3, S4, S5, N1, N2, N3, N4, N5	South, North	21	1/0.75	18/6	600	Lightly boiled spinach	Uppsala	Eggs collected
Lindgren and Laurila (2010)	2004	Jukkasjärvi	North	18	10/80	18/6	5	Lightly boiled spinach	Uppsala	Eggs collected
Lind and Johansson (2007, 2011), Lind et al. (2011)	2005	Ahällan, Algrundet, Bredskär, Buten, Gåshällan, Sävar, Grisselögern, Lillklyvan, Tärnögern, Svart Lass, Vitskär	Mid North	22	1/0.75	18/6	400	2:1 mixture of rabbit pellets and aquarium fish flakes	Umeå	Eggs collected
Lind and Johansson (2007, 2009, 2011), Lind et al. (2011)	2006	Ahällan, Algrundet, Bredskär, Buten, Gåshällan, Grisselögern, Lillklyvan, Sävar Tärnögern, Svart Lass, Vitskär	Mid North	22	1/0.75	18/6	360	1:1 mixture of rabbit pellets and aquarium fish flakes	Umeå	Eggs collected
Orizaola et al. (2013)	2008	Allskog Maryds, Jukkasjärvi, Björkliden	South, North	19	10/10	16/8	20	Lightly boiled spinach	Uppsala	Eggs collected
Johansson et al. (2010)	2008	Bredskär, Lillhaddingen, Stora Fjäderagg, Storhaddingen, Ahällan, Algrundet, Fjärdgrund, Sävar Tärnögern	Mid North	22	1/0.75	18/6	160	1:1 mixture of rabbit pellets and aquarium fish flakes	Umeå	Eggs collected

### Data

We searched for published studies, in which either freshly laid eggs or reproducing adults were collected, across Sweden and northern Finland. In total, data for *R. temporaria* were collected over 11 years between 1998 and 2009, from 57 sampling occasions at 26 different populations (breeding ponds), located between latitudes 55° N and 69° N. Hence, in our study, each data point refers to a specific year and population sampling location. In total, we collected 11 data sets from 14 published studies (Table 1). In these studies, either parents were captured at breeding sites, and subsequently artificially crossed to produce offspring or alternatively, freshly laid eggs were collected in the wild and brought to the laboratory (Table 1). All eggs and larvae were then raised under standardized laboratory conditions until metamorphosis, and larval period, mass at metamorphosis and growth rate were all estimated (See Online Resource 1 for more details on data collection and calculation). All studies included in this analysis were either conducted at Uppsala University or at Umeå University in Sweden (See Table 1 and Fig. S2, Online Resource 2), and all larvae were kept under relatively similar conditions. In short, the larvae were raised in plastic containers (see Table 1 for details), fed ad-libitum with spinach or commercial fish flakes and rabbit pellets, kept at temperatures of 18–22 °C, photoperiod of 16 h light/8 h dark to 18 h light/6 h dark, and without any cues from the external natural environment. The lack of cues from the natural environment was especially important, as conditions like temperature, photoperiod, and food variation can influence larval development rates.

### Variables of interest

We were primarily interested in larval period, measured as the number of days between Gosner stage 25 (disappearance of gills) and 42 (emergence of forelimbs) (Gosner 1960), as this variable has the strongest connection to our prediction, that decreased length of growing season correlate with increased rates of larval development. Although mass at metamorphosis and growth rate (measured as the ratio between mass at metamorphosis and larval period) would also be interesting to investigate, unfortunately there were only limited observations for each of the latitudes (between 2 and 10) that reported both mass at metamorphosis, or growth rate, and their standard errors. In addition, we obtained data on two predictors: (1) *reproductive delay *the period between the mean first day of spring for a given location and reproductive event in the parental generation; the first day of spring is the first of seven consecutive days with average temperatures over 0 °C, based on 1961–1990 average (SMHI 2017, see Online Resource 2, Fig. 2S); (2) *latitude* with four levels corresponding to four latitudinal groups, ordered from: southern (55° N–57° N, *n* = 14), mid-southern (59° N–61° N, *n* = 2), mid-northern (63° N–64° N, *n* = 28), to northern (67° N–70° N, *n* = 15) (see Fig. S2), as the populations of *R. temporaria* were sampled across a latitudinal gradient in Sweden and northern Finland (Online Resource 2, Fig. S2). There were only two observations from mid-southern populations (Fig. S2), so these two data points were not used in the analysis. To account for any differences in experimental procedures between studies, we included laboratory location, laboratory temperature, photoperiod, food, and larval density as additional predictors in our analyses.

### Models

#### Bayesian models

We used Bayesian mixed-effect models, with the mean larval period for a given population in each study as a single response data point. A Gaussian error distribution was assumed in the model, and this assumption was visually confirmed from plots of the data. Following a meta-analytical approach, sampling error variance of the estimated mean was implemented in the model, calculated as the squared standard error (Hadfield 2010). Study ID, population, and the year × population interaction were added as random effects. The population term referred to the specific population (breeding pond) sampled in any given study (Table 1). To avoid an over-parameterized model, while still assessing the effect of potentially confounding variables, we used a combination of a pre-set model (the variables for which we had an explicit biological interest in), and a forward model selection approach (for the variables that we wanted to control for—which were related to lab specific rearing conditions). The explanatory variables for which we had explicit biological interest in (reproductive delay, latitude, and their interaction) were consequently added as fixed effects to all models we assessed. For the additional variables, that we had no explicit interest in but that potentially could confound our results, we used a forward selection approach. We used Deviance Information Criterion (*DIC*, Spiegelhalter et al. 2002) to select the main model. *DIC* is a hierarchical modeling generalization of Akaike Information Criterion (*AIC*). Specifically, laboratory temperature, photoperiod, food, larval density, and laboratory location were included in separate models, including their interaction with latitude. These additional variables were removed if they did not improve the fit of the model (judged as a delta *DIC* > 1). Each of these models hence contained the added variable that we wanted to control for, its interaction with latitude, and the parameters that we had biological interest in: delay, latitude, and their interaction, as fixed effects. In the cases were the inclusion of a variable and its interaction with latitude improved the fit of the model, we further assessed the inclusion of the interaction. The final model, chosen on the basis of its *DIC* value, was a model containing delay, latitude, their interaction, as well as laboratory rearing temperature, as fixed effects (see Online Resource 3, Tables S3.1–S3.7 for model formulas, *DIC* values, and results of all the models). Inclusion of photoperiod and study location improved the fit only marginally (delta *DIC* < 1), so these variables were not included in the final model. Southern population was set as a reference in the main model, with all other populations being compared with this population. To assess the latitude specific value of the effect of breeding delay (as indicated by the interactions between delay and latitude), we fit different versions of the same model, where the different models (otherwise identical), had a different reference latitude. We used MCMCglmm (Hadfield 2010) mixed models, in R version 3.6.0 (R Development Core Team 2019).

We chose priors that should be non-informative for the result. Hence, a flat, Gaussian prior with mean zero and a variance of 10^10^ was used for the fixed effects, and locally non-informative, inverse Wishart priors, with prior variance estimate set to one fourth of the total phenotypic variance (hence having a prior belief of equal weights to all variance components), and belief parameter set to 0.002, was used for the random effects. Both represent little prior knowledge (see Online Resource 4 for more details on the specification of priors). We run at least 2 × 10^6^ iterations, a burn-in of 5 × 10^4^, and a thinning interval of 2000, which resulted in effective sampling size of 1000. The predictors were centered to a mean of 0 in order to give model estimates at the mean predictor values (Schielzeth 2010). To increase comparability, we estimated standardized regression coefficients by scaling predictors to a standard deviation of 1. We diagnosed posterior distributions and model convergence by running three parallel chains using the Gelman–Rubin convergence criterion, and the upper 97.5 quantile of the Gelman–Rubin test statistic was below 1.2 in all cases. All autocorrelations were within the interval − 0.1 and 0.1. A precision plot did not show any major asymmetry, suggesting that there was no publication bias in our data (Online Resource 5, Fig. S5).

## Results

### Larval period length at different latitudes

Our results confirmed that the larval period of *R. temporaria* is shorter in northern compared to southern populations (*β* = − 2.36; 95% CI − 4.41, − 0.359; *P*_MCMC_ < 0.02).

### Reproductive delay and latitude interaction

**Fig. 1:**
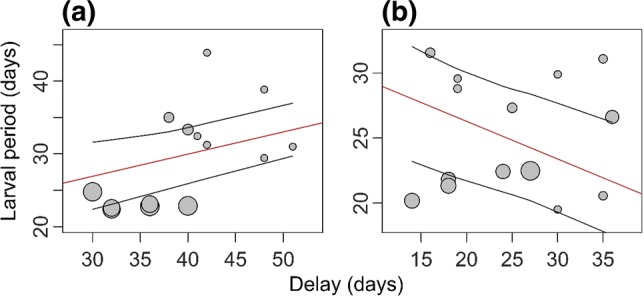
The relationship between reproductive delay and larval period in **a** southern, and **b** northern populations of *R. temporaria*. The regression lines (red) are placed between the 95% confidence interval range lines (black), and are based on estimates from the model containing delay, latitude, and their interaction as fixed effects. Area of the points corresponds to the precision of the estimates (1/SEM_estimate_)

The slope of the reproductive delay differed between the southern and mid-northern populations (*β* = − 3.38; 95% CI − 5.48, − 1.29; *P*_MCMC_ = 0.002) and between the southern and northern populations (*β* = − 4.35; 95% CI − 5.78, − 2.93; *P*_MCMC_ < 0.001). Larval period increased significantly with breeding delay in the southern populations (*β* = 2.4; 95% CI 0.979, 3.84; *P*_MCMC_ < 0.001; Fig. 1a) and decreased in the northern populations (*β* = − 1.94; 95% CI − 2.81, − 1.10; *P*_MCMC_ < 0.001; Fig. 1b).

## Discussion

We investigated the effects of reproductive delay in the parental generation, on the larval period in *R. temporaria* frog populations, across a latitudinal gradient in Sweden. We found that breeding delay affected offspring traits. In the north, larval period decreased with increasing reproductive delay, while the opposite was observed for the southern populations. The lack of any external environmental cues for larvae under common-garden rearing, suggests a parental origin for observed changes in larval life history traits.

Interestingly, the effect of breeding delay was of similar magnitude, but in different directions for the southern and northern populations. Specifically, a higher reproductive delay induced shorter larval period in the north, but longer larval periods in the south. In accordance with previous experimental research, which artificially postponed reproduction on mid-southern populations of *R. arvalis* and *R. temporaria* (Lindgren and Laurila 2010; Richter-Boix et al. 2014; Orizaola et al. 2016), we found a decreased larval period in response to later reproduction, in northern populations, which suggests an adaptive response to a shortened growing season. Moreover, similar to our study, Orizaola et al. (2013) found that northern populations of *R. temporaria* developed faster during a year with a shorter growing season, and the magnitude of change was comparable with our study (compare Fig. 1b in the present study with Fig. 3a, b in Orizaola et al. 2013). These results indicate that parental effects in vertebrates from time-constrained environments may act to further decrease their already short development times.

Southern *R. temporaria* populations, on the other hand, had longer larval developmental times as a response to increasing breeding delay. If the length of the growing season is sufficient, even after a delay, there may not be any reasons to expect that a breeding delay would decrease larval period. However, this does not explain the significant increase in breeding delay that we observed in our study. Previous studies have shown that delayed *R. temporaria* and *R. arvalis* tadpoles, increase their development rates, but also lower antipredator defenses (Orizaola et al. 2013, 2016), which is likely to increase predator-mediated mortality. Moreover, it has been shown that in the wild, tadpoles from southern populations of *R. temporaria* experience higher predator densities than tadpoles from northern populations (Laurila et al. 2008). Indeed, the lower predator densities and lower predator activity due to lower water temperatures may facilitate higher developmental rates in wild northern populations, as fast growth rate trades off with other life history traits and requires higher foraging activity that may lead to increased mortality (Gotthard et al. 1994; Gotthard 2000, 2008; Laurila et al. 2006, 2008). Hence, the increase in the larval period in the southern populations observed in our study could be an indication of such an adaptive trade-off, i.e., the breeding delay affects larval period in the southern populations by modulating a third, unmeasured variable, such as growth rate/mortality.

Predation on amphibians may be size-, or stage-specific (Werner 1986; Kaplan 1992; Urban 2007), meaning that under certain conditions, longer or shorter larval periods may facilitate escape from tadpole stage-specific predators, like dragonfly larvae, and consequentially be evolutionary advantageous. Therefore, increased larval period by southern *R. temporaria* populations could be adaptive despite the delay in reproduction, since longer larval periods may be caused by lower tadpole activity, as a predator avoidance strategy. In addition, as ectotherms, dragonfly larvae are more active at higher temperatures, and water in the southern locations will likely become warmer sooner than in the north. Therefore, the appearance of tadpoles in these ponds will coincide with already highly active dragonfly larvae. Tadpoles are exposed for a longer time to highly active dragonfly larvae in the south, compared to in the north, when the breeding season has been delayed. As a consequence, southern populations could show a stronger response of reduced activity and development rate to avoid more active predators in a late reproductive season.

Studies on other organisms have shown latitudinal specific parental effects on growth and performance, and these effects often result in evolutionary trade-offs (e.g., insects: Mousseau and Dingle 1991; fish: Johnston and Leggett 2002; reptiles: Sinervo 1990; humans: Staples et al. 2010). For example, lizards from northern latitudes produce smaller eggs that develop faster, hatchlings that grow faster, but also juveniles with slower sprint speeds, potentially making them more vulnerable to predators (Sinervo 1990). However, the production of small eggs in northern populations could still maximize fitness, if larger eggs experienced higher mortality, as selection may maximize maternal fitness at the cost of offspring fitness (Marshall and Uller 2007). For example, females that reproduce multiple years may limit investment in eggs, and produce low-quality eggs in case of temporarily unfavorable environmental conditions. In our study, this would mean that females produced slow-developing eggs in years with delayed reproduction, if these years were perceived as having unfavorable environmental conditions.

A caveat to our study is that our data was extracted from previous studies, rather than being collected with a fully standardized methodology, as would be the case for experimental data. Hence, some of the variables in our data, such as laboratory temperature, food and latitude, were somewhat confounded. Most of the confounds in our data are caused by data being included from two research groups, one in Uppsala, and one in Umeå (both in Sweden). However, we do not see this as being problematic in our study, because our main results and conclusions were based on differences between the northern and southern populations, which were investigated only by the Uppsala based research group. Further, in addition to parental effects, alternative explanations of our results are possible. For example, delayed breeding could result in carry-over effects associated with parental physiological stress. In such a case, southern *R. temporaria* populations could be less able to cope with conditions associated with reproductive delay than northern populations, and produce slower developing offspring. Mother–offspring stress hormone transfer through egg yolk is a well-known phenomenon (Sheriff and Love 2013), and previous studies have suggested that climate and weather conditions may influence stress hormone levels in vertebrates (Sheriff et al. 2012). Moreover, previous research on *R. temporaria* found that southern populations had higher corticosterone levels in response to stress than northern populations, which in turn, decreased growth and development (Dahl et al. 2012). Therefore, it is possible that egg-mediated hormonal changes in offspring could influence the larval period in southern populations of *R. temporaria.*

## Conclusions

Our results suggest that local adaptation to a short growing season has resulted in potentially adaptive parental effects of delayed breeding time in northern *R. temporaria* populations. We suggest that many of the latitudinal differences in parental effects documented may potentially be explained either by differences in life-history trade-offs, or by the evolution of sensitivity to latitude-specific cues, presenting a fruitful avenue for further research.

## Electronic supplementary material

Below is the link to the electronic supplementary material.

Supplementary file1 (PDF 447 kb)

Supplementary file2 (XLS 56 kb)
