# Supplementary material for: Parental effects influence life history traits and covary with an environmental cline in common frog populations
Source: Oecologia. 2020 Apr 10;192(4):1013–22. doi: 10.1007/s00442-020-04642-8 (PMC7165185; doi:10.1007/s00442-020-04642-8)
Supplement: Supplementary file 1 — Supplementary file1 (PDF 447 kb) [file 442_2020_4642_MOESM1_ESM.pdf]

ONLINE RESOURCES

Parental effects influence life history traits and covary with an environmental cline in common frog populations

Piotr K. Rowiński, Anssi Laurila, Karl Gotthard, Will Sowersby, Martin I. Lind, Alex Richter-Boix, Simon Eckerström-Liedholm, Björn Rogell

Authors for correspondence:

Piotr K. Rowiński

Email: Piotr.Rowinski@zoologi.su.se

Björn Rogell

Email: Bjorn.Rogell@slu.se

Present address:

Department of Aquatic Resources, Institute of Freshwater Research, Swedish University of Agricultural Sciences, Drottningholm, 17893 Sweden

Online Resource 1: additional information on study species and data collection.

Common frog (*Rana temporaria*) is an explosive breeder, laying eggs in shallow waters. The species is ecologically similar to the moor frog (*R. arvalis*) with a comparable diet (Stojanova and Mollov; 2008). *R. temporaria* can be encountered from northern France in the west to Ural Mountains in the East, and from northern Spain and the Balkans in the South to the northern Skandinavia in the North (Kuzmin et al. 2009). In Sweden, *R. temporaria* populations are encountered in more northern, mountainous, and dry habitats than *R. arvalis.*

Data collected from the studies:

- Study
- Population
- Collection year
- Reproduction date
- Larval period and its standard error
- Mass at metamorphosis and its standard error
- Growth rate and its standard error
- Sample size of larvae raised to metamorphosis
- Laboratory temperature
- Larvae density under laboratory conditions
- Food in laboratory conditions
- Photoperiod in laboratory conditions
- Latitude and longitude for the sampled populations. Sampling coordinates were subsequently transformed from degrees and minutes to decimal degrees with the help of Earth Point web site (Earth Point 2017).

We collected data on First day of spring for given population coordinates, from the Swedish Meteorological and Hydrological Institute (SMHI 2017, Figure S1):

From the collected data we calculated Reproductive delay; a period between the first day of spring and reproductive event in maternal generation. The first day of spring is the first of seven consecutive days with average temperatures over 0°C, based on 1961-1990 average (SMHI 2017, see Online Resource 2).

Online Resource 2: First day of spring and sampling locations

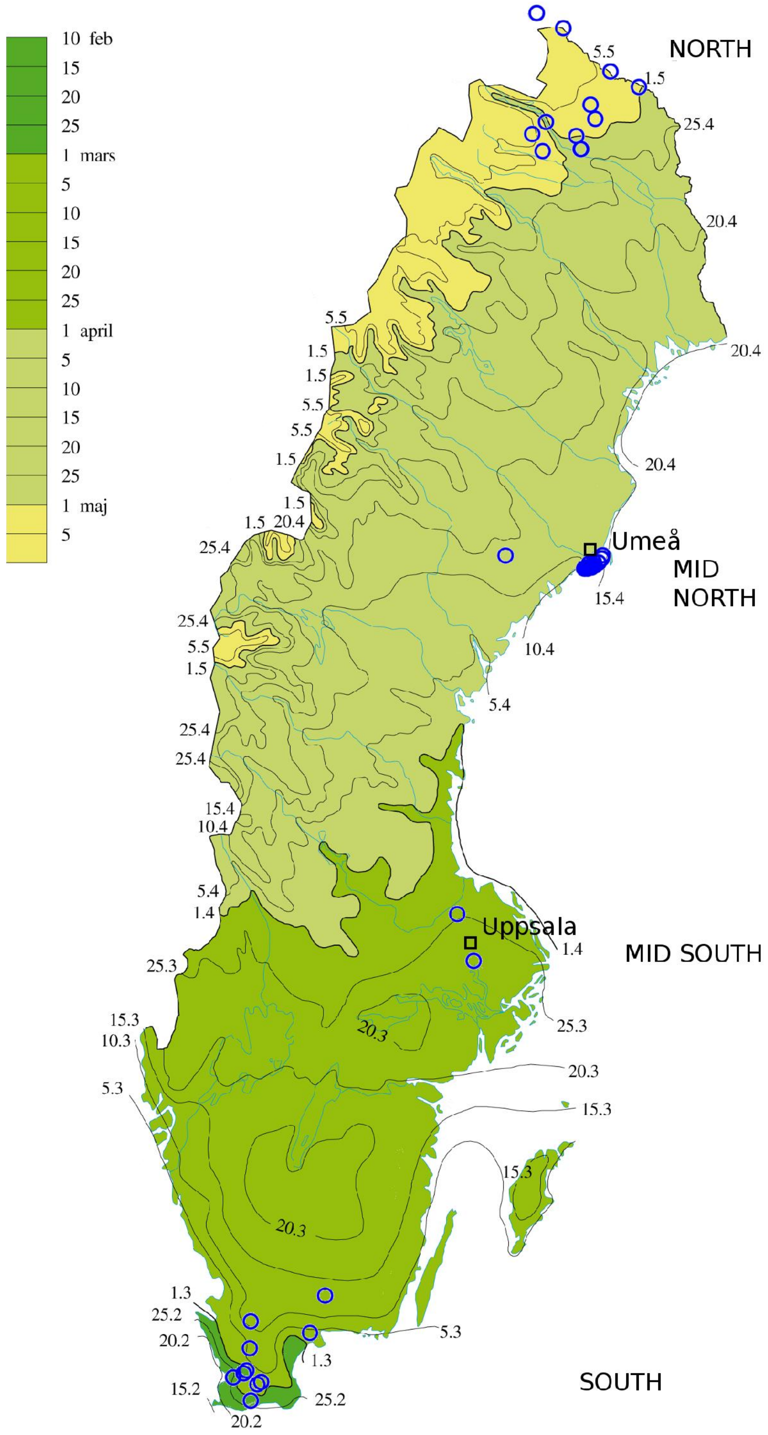

Figure S2. The first day of spring across Sweden, based on 1961-1990 averages (SMHI 2017a), and sampling areas for *R. temporaria* (circles), included in the analysis as four latitude groups (south, mid south, mid north, and north). The above map was used to estimate the first day of spring for each population sampled that was used in the present study. Laboratory locations where the common garden experiments were performed (Uppsala and Umeå) are indicated by black squares.

Online Resource 3: The models

Models investigating relationship between reproductive delay and larval period

Table S3.1. Model 1. larv.per~delay*latitude, DIC = 230.0772

	Mean	Lower 95% CI	Upper 95% CI	pMCMC
**Fixed effects**				
Intercept	27	23.1	31.3	0.001
delay	2.56	0.605	4.3	0.006
mid.north	1.24	-3.05	5.67	0.538
north	-2.35	-5.45	0.564	0.116
delay:mid.north	-3.4	-6.25	-0.245	0.034
delay:north	-4.59	-6.47	-2.72	0.001
**Random effects**				
study	23.5	8	61.4	NA
population	0.0978	0.00345	0.496	NA
year:population	0.0969	0.00302	0.507	NA

Table S3.2. Model 2, food. larv.per~delay*latitude+food*latitude, DIC = 232.901

	Mean	Lower 95% CI	Upper 95% CI	pMCMC
**Fixed effects**				
Intercept	25.3	13.5	37	0.001
delay	2.53	0.7	4.44	0.014
mid.north	2.58	-2.12	7.52	0.284
north	-2.32	-5.11	0.694	0.12
food2.1	-2.72	-14.9	10.4	0.608
food3.1	0.476	-12	13.9	0.932
food.spinach	3.81	-7.56	15.8	0.472
delay:mid.north	-3.48	-6.46	-0.426	0.036
delay:north	-4.5	-6.51	-2.47	0.002
north:food3.1	-0.334	-3.58	3.22	0.876
**Random effects**				
study	28.8	7.81	81.4	NA
population	0.104	0.00278	0.553	NA
year:population	0.0966	0.0028	0.535	NA

Table S3.3. Model 3, density. larv.per~delay*latitude+density*latitude, DIC = 232.627

	Mean	Lower 95% CI	Upper 95% CI	pMCMC
**Fixed effects**				
Intercept	34.3	21.6	46.4	0.001
delay	2.44	0.434	4.46	0.014
mid.north	6.28	-10.3	24.6	0.482
north	-4.62	-15.1	5.6	0.368
density	-5.83	-16.2	5.28	0.254
delay:mid.north	-3.46	-6.59	-0.501	0.024
delay:north	-4.48	-6.29	-2.53	0.001
mid.north:density	-4.9	-22.8	11.3	0.564
north:density	1.74	-5.58	9.1	0.646
**Random effects**				
study	23.7	6.96	63.8	NA
population	0.104	0.00313	0.601	NA
year:population	0.104	0.00318	0.564	NA

Table S3.4. Model 4, photoperiod. larv.per~delay*latitude+photoperiod*latitude, DIC = 229.2032

	Mean	Lower 95% CI	Upper 95% CI	pMCMC
**Fixed effects**				
Intercept	25	19.8	29.6	0.001
delay	0.945	-1.59	3.34	0.44
mid.north	1.12	-5.99	8.62	0.736
north	-2.73	-5.99	0.0189	0.056
photoperiod.16_8	7.83	0.512	14.8	0.044
delay:mid.north	-1.89	-5.25	1.6	0.284
delay:north	-2.82	-5.62	-0.0393	0.05
mid.north:photoperiod.16_8	-1.55	-10.3	7.76	0.7
north:photoperiod.16_8	-2.93	-6.99	0.723	0.13
**Random effects**				
study	15.7	3.79	45.9	NA
population	0.113	0.00311	0.663	NA
year:population	0.101	0.00299	0.58	NA

Table S3.5. Model 5, study location. larv.per~delay*latitude+study location, interaction of study location with latitude not estimated due to too low observation number, DIC = 229.9725

	Mean	Lower 95% CI	Upper 95% CI	pMCMC
**Fixed effects**				
Intercept	23.3	15.9	31.6	0.001
delay	2.53	0.734	4.43	0.01
mid.north	2.64	-2.25	7.73	0.312
north	-2.44	-5.33	0.334	0.108
Uppsala	4.41	-3.33	12.8	0.236
delay:mid.north	-3.53	-6.65	-0.542	0.022
delay:north	-4.6	-6.46	-2.83	0.001
**Random effects**				
study	24	7.85	67.8	NA
population	0.106	0.00284	0.671	NA
year:population	0.0863	0.00307	0.465	NA

Table S3.6. Model 6, temperature interaction. larv.per~delay*latitude+lab temperature*latitude, DIC = 53.82122

	Mean	Lower 95% CI	Upper 95% CI	pMCMC
**Fixed effects**				
Intercept	25.3	21.9	29	0.001
delay	2.38	1.13	3.79	0.001
mid.north	3.02	-1.98	8.01	0.202
north	-2.31	-4.09	-0.301	0.026
temp.lab	-3.57	-4.66	-2.49	0.001
delay:mid.north	-3.38	-5.55	-1.52	0.001
delay:north	-4.33	-5.77	-2.99	0.001
mid.north:temp_lab	0.459	-2.48	3.2	0.708
north:temp_lab	0.101	-0.923	1.22	0.848
**Random effects**				
study	19.6	5.96	52.8	NA
population	0.375	0.00435	1.48	NA
year:population	0.37	0.00463	1.33	NA

Table S3.7. Model 7, the chosen model, temperature without interaction. larv.per~delay*latitude+lab temperature, DIC = 48.05925. The chosen model with the lowest DIC value.

	Mean	Lower 95% CI	Upper 95% CI	pMCMC
**Fixed effects**				
Intercept	25.5	22.3	28.9	0.001
delay	2.4	0.979	3.84	0.001
mid.north	2.59	-0.757	5.87	0.102
north	-2.36	-4.41	-0.359	0.02
temp.lab	-3.47	-4.41	-2.58	0.001
delay:mid.north	-3.38	-5.48	-1.29	0.002
delay:north	-4.35	-5.78	-2.93	0.001
**Random effects**				
study	17.1	6.15	43.2	NA
population	0.339	0.00456	1.24	NA
year:population	0.387	0.00457	1.29	NA

Online Resource 4: Specification of priors for random effects

Prior formula:

prior <- list(R = list(V = V1*/4, nu = 0.002),

G = list(G1 = list(V= V1/4, nu = 0.002),

G2 = list(V= V1/4, nu = 0.002),

G3 = list(V= V1/4, nu = 0.002)))

*V1 is variance in larval period across all the populations

Online Resource 5: Precision plot

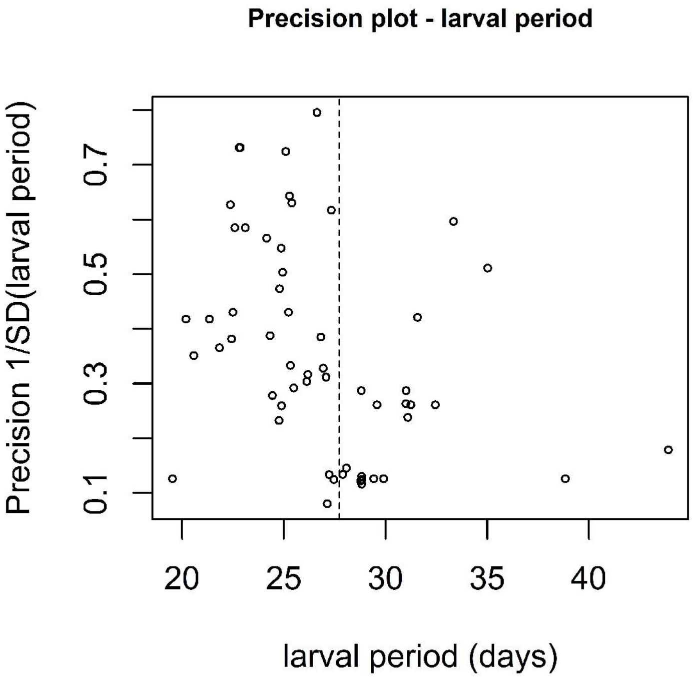

Figure S5. Precision plot with the vertical line corresponding to posterior mean estimate, showing larval period estimates on the x axis and precision (1/standard deviation of the larval period) on y axis.

REFERENCES:

Earth Point 2017 Earth Point, tools for Google Earth. URL: http://www.earthpoint.us/Convert.aspx, last accessed 2017-12-18.

Kuzmin S, Ishchenko V, Tuniyev B, Beebee T, Andreone F, Nyström P, Anthony BP, Schmidt B, Ogrodowczyk A, Ogielska M, Bosch J, Miaud C, Loman J, Cogalniceanu D, Kovács T, Kiss I 2009 *Rana temporaria*. (errata version published in 2016) The IUCN Red List of Threatened Species 2009b: e.T58734A86470817. URL: http://dx.doi.org/10.2305/IUCN.UK.2009.RLTS.T58734A11834246.en. Last accessed 2017-12-17.

SMHI 2017. Swedish Meteorological and Hydrological Institute. URL: https://www.smhi.se/kunskapsbanken/meteorologi/var-1.1080. Last accessed 2017-12-18.

Stojanova AM, Mollov IA (2008) Diet and trophic niche overlap of the moor frog (*Rana arvalis* Nilsson, 1842) and the common frog (*Rana temporaria* L., 1758) from Poland. In Anniversary Scientific Conference of Ecology, Proceedings (Vol. 181190).
